# CCDN-DETR: A Detection Transformer Based on Constrained Contrast Denoising for Multi-Class Synthetic Aperture Radar Object Detection

**DOI:** 10.3390/s24061793

**Published:** 2024-03-11

**Authors:** Lei Zhang, Jiachun Zheng, Chaopeng Li, Zhiping Xu, Jiawen Yang, Qiuxin Wei, Xinyi Wu

**Affiliations:** 1 School of Ocean Information Engineering, Jimei University, Xiamen 361021, China; 202111810011@jmu.edu.cn (L.Z.); jchzheng@jmu.edu.cn (J.Z.); xzpxmu@gmail.com (Z.X.); 202312854003@jmu.edu.cn (J.Y.); 202121306003@jmu.edu.cn (X.W.); 2 Fujlan Electronic Port Co., Ltd., Xiamen 361000, China; weiqx@xpgco.com.cn

**Keywords:** detection transformer, SAR, object detection, deep learning

## Abstract

The effectiveness of the SAR object detection technique based on Convolutional Neural Networks (CNNs) has been widely proven, and it is increasingly used in the recognition of ship targets. Recently, efforts have been made to integrate transformer structures into SAR detectors to achieve improved target localization. However, existing methods rarely design the transformer itself as a detector, failing to fully leverage the long-range modeling advantages of self-attention. Furthermore, there has been limited research into multi-class SAR target detection. To address these limitations, this study proposes a SAR detector named CCDN-DETR, which builds upon the framework of the detection transformer (DETR). To adapt to the multiscale characteristics of SAR data, cross-scale encoders were introduced to facilitate comprehensive information modeling and fusion across different scales. Simultaneously, we optimized the query selection scheme for the input decoder layers, employing IOU loss to assist in initializing object queries more effectively. Additionally, we introduced constrained contrastive denoising training at the decoder layers to enhance the model’s convergence speed and improve the detection of different categories of SAR targets. In the benchmark evaluation on a joint dataset composed of SSDD, HRSID, and SAR-AIRcraft datasets, CCDN-DETR achieves a mean Average Precision (mAP) of 91.9%. Furthermore, it demonstrates significant competitiveness with 83.7% mAP on the multi-class MSAR dataset compared to CNN-based models.

## 1. Introduction

Synthetic aperture radar (SAR) is an active remote sensing technology that provides high-resolution, all-weather and weather-independent ground imaging, achieved by attaching a radar system to a mobile platform and utilizing the moving synthetic aperture method. This technology is invaluable for Earth observation, aiding in the monitoring of surface deformation, surveillance of geological hazards, and tracking changes in snow and ice cover. The all-weather capability and high-resolution positioning of SAR are essential for detecting significant surface changes. Beyond Earth observation, SAR is critical in military reconnaissance, environmental monitoring, and numerous other fields. SAR images, which are typically displayed in black and white, represent radar signal strength and offer a contrast to color optical images. However, their strong reflections and indistinct edges can make object detection challenging. Traditional methods, such as using Constant False Alarm Rate (CFAR) to adapt detection thresholds based on background levels, may face difficulties with complex SAR images due to their fixed settings [1].

**Figure 1:**
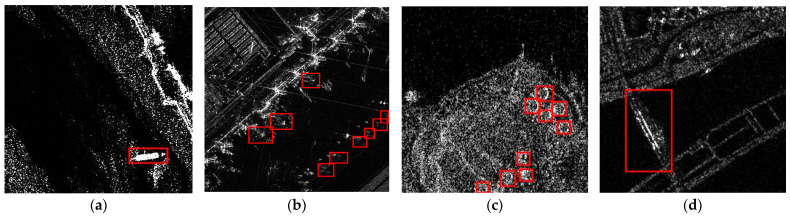
SAR imaging differences for objects of different categories. The red box shows the real annotation information of the corresponding objects. SAR ship targets exhibit strong interference from nearby coastal backgrounds, aircraft targets have discrete features and incomplete structures [23], and oil tank targets are closely spaced, making them prone to omission or false detection. These challenges underscore the complexities in multi-class SAR object detection. (**a**) Ship. (**b**) Aircraft. (**c**) Tank. (**d**) Bridge.

In recent years, deep learning-based object detection methods have continuously made progress, and researchers are increasingly exploring their applications in the SAR domain. SAR target detection algorithms based on CNNs can be divided into two-stage detectors and single-stage detectors. Two-stage methods require feature extraction and classification regression tasks to be performed on multiple candidate regions, with Faster R-CNN [2] being a representative method. Single-stage detectors, on the other hand, do not generate candidate regions but predict categories and bounding boxes directly in the image. Typical methods include YOLO [3,4,5], RetinaNet [6], and SSD [7], among others. Researchers have attempted to adapt and apply these methods to SAR target detection by making specific improvements. These improvements mainly include using stronger backbone networks [8,9], setting up multi-scale FPN layers [10,11], and designing loss functions more suitable for SAR tasks [12,13]. Meanwhile, real-time SAR target detection schemes [14,15,16] are also gradually developing, providing references for the practical application of SAR detection and recognition. Moreover, the transformer [17] architecture has demonstrated remarkable efficacy in optical image recognition by leveraging the global modeling capabilities of its self-attention mechanism. This success has prompted efforts to integrate transformer structures into the SAR imaging domain. For instance, CRTransSar [18] adopts the Swin Transformer [19] as the backbone network and introduces a cross-resolution attention enhancement module to fuse multiscale features, achieving exceptionally high detection accuracy. Sun et al. [20] enhanced the feature fusion layer of YOLO V5 [21] with self-attention, establishing connections with global information, and thereby improving the detection performance of small-scale SAR targets. Zha et al. [22] replaced the last convolutional block of a backbone network with a transformer structure, enriching the contextual semantic information and achieving successful benchmark results. Notably, although certain approaches utilize a transformer structure to replace the CNN backbone, this methodology is not end-to-end. The inference process of the model is still influenced by nonmaximal inhibition, limiting the complete exploitation of the capacity of the transformer for encoding and decoding global information. Additionally, as shown in Figure 1, SAR targets of different categories exhibit differences in imaging characteristics, which pose challenges for multi-class SAR object detection. To address these issues, our proposed solution is to use the DETR framework for end-to-end multi-class SAR object detection.

The detection transformer [24] (DETR) redefines the object detection problem as a sequence-to-sequence task consisting of three main components: a pretrained backbone feature extraction network and encoders and decoders equipped with self-attention mechanisms. The backbone network is used to extract features and transform them into vectors that encode spatial information in the image through position coding. The encoder processes feature vectors and position coding to extract deep-level features. In the decoder stage, the output of the encoder is decoded by N learnable queries, resulting in N feature vectors that predict the class of the object and the location of the instance box. DETR’s encoder and decoder of DETR typically include multiple corresponding coding and decoding layers to improve the overall performance. Unlike CNN-based object detectors, DETR eliminates the need to manually generate components such as ROI and post-processing steps such as NMS. Instead, it directly predicts the location of the target in the image through the transformer’s self-attention mechanism, enabling end-to-end inference. This approach provides a new perspective on object detection in SAR imagery. 

This study draws on the methodology of DINO-DETR, whose introduced denoising mechanism significantly reduces the training difficulty of DETR models and markedly improves the model’s performance in detecting small objects. Building on this approach, we propose CCDN-DETR for performing end-to-end SAR object detection. Specifically, we introduced a cross-scale coder to replace the original self-attention-based multilayer coder. This novel coder extends the resolution scale by leveraging the inductive bias of the convolution and the global modeling capability of the transformer. It aims to facilitate intralayer information encoding and interlayer information fusion, thereby enhancing localization capabilities in the presence of substantial differences in the target scale. To address the limitations of SAR data, characterized by its finite nature and high signal-to-noise ratio, and to enhance the effectiveness of multi-category SAR object detection, we incorporated constrained contrastive denoising during the decoder training phase. This involved leveraging both real data and noise data to improve the training process. Unlike DION-DETR [25], we constrain the real data within a specific range to delineate a clear boundary contour. Furthermore, we redefined the initialization scheme for instance queries in the decoder layer. We introduced IoU loss to guide the model in selecting features with high-precision predicted boxes and high classification scores. Additionally, we designed a more potent decoder layer. To demonstrate the effectiveness of our approach, we conducted experiments by consolidating the SSDD [26], HRSID [27], and SAR-AIRcraft datasets [28] into a unified dataset. This amalgamated dataset comprises two target categories (ships and aircraft) and 35,772 image samples. Furthermore, we report the performance of the proposed method on a multi-class MSAR dataset [18]. 

The contributions of this study can be succinctly summarized as follows:

(1). We propose the adoption of a cross-scale coder instead of the original multilayer transformer coder. This innovative approach expands the information scale, facilitating profound information modeling and fusion for the detection of SAR targets with significant scale differences.
(2). The selection scheme for input DETR decoder queries is improved to guide the model to select features with high classification and IoU scores. Additionally, we designed a more potent decoder to further optimize the recognition efficiency.
(3). To address the finite nature of SAR data and their high signal-to-noise ratio, we introduced constrained contrast denoising in the training phase. Real and noise labels were integrated into the decoder layer for contrast training. The real labels were confined within a controlled range, aiding the network to concentrate on clear boundary contours.
(4). We present an end-to-end SAR object-detection solution called CCDN-DETR. This novel multiclass detection transformer is specifically tailored to the SAR domain. The effectiveness of DETR for SAR object detection was demonstrated through experiments conducted on two extensive multi-category datasets.

## 2. Related Studies

### 2.1. SAR Object Detection Based on CNN

Advances in synthetic aperture radar (SAR) object detection often build upon the developments in optical object detection technology. Current CNN-based methods focus on innovative model structures, and on the basis of popular detectors such as Faster R-CNN [2], YOLO [3,4,5], SSD [7], and RetinaNet [6], they achieve information fusion through the design of new network architectures to fully leverage the inductive bias capabilities of convolutions. Representative methods include the integration of attention mechanisms (SE [29]) into the feature extraction part of the backbone network by Hou et al. [18], providing additional attention to the detection regions; L-YOLO [30] achieves a lightweight, efficient network structure by simplifying convolution operations and proposes a k-means algorithm for clustering anchor boxes; Miao T et al. [8] improved model accuracy by adjusting the backbone network and applying channel and spatial attention. Additionally, integrating SAR-based prior knowledge [31,32] with deep learning helps to provide more stable detection results. Moreover, some specifically optimized loss functions [12,13] have also gained attention and have been demonstrated to perform well in SAR object detection tasks. Despite the good detection results achieved by existing CNN methods, they have overlooked the advantages of self-attention in modeling long-distance information. Our proposal is to treat the transformer itself as a detector, capturing global dependencies to achieve better detection results.

### 2.2. Detection Transformer (DETR)

DETR [14], introduced in 2020, is an innovative end-to-end object detection framework based on the transformer architecture. A typical DETR structure consists of three parts: the backbone network, the encoder, and the decoder. The backbone network and the encoder are used for feature extraction, while the decoder predicts the categories and bounding boxes of the objects. DETR employs a set prediction mechanism, using the Hungarian algorithm to match the predicted set of objects with the real set of labels, ensuring that each predicted object is paired with a unique real object, rather than generating a large number of candidate boxes for screening. Unlike traditional CNN-based object detection methods, DETR does not require additional post-processing steps such as ROI pooling and NMS. This marks a paradigm shift in object detection from a multi-step process to an end-to-end solution. DETR simplifies the process of object detection and has achieved significant results in optical object detection [33,34,35]. Additionally, researchers have also explored the application of DETR in SAR object detection. Notable works include OEGR-DETR [13], which proposes an OEM module and GRC loss for enhancing the localization of rotated objects; Chao Ma [34] et al. propose cylinder IOU and incident angle priors for end-to-end 3D SAR object detection; TSDet [35] uses an enhanced attention module for precise identification of SAR ship targets. These works validate the effectiveness of DETR for SAR object detection, and our method will further explore the performance of DETR in multi-class SAR object detection.

### 2.3. Multiclass SAR Datasets

An abundant dataset for training is a crucial prerequisite for advancing computer vision technology, and this holds true in the domain of SAR object detection. The release of the first publicly available SAR object detection dataset, SSDD [26], in 2017 garnered significant attention from researchers and set a benchmark for SAR object detection. Subsequently, more and more high-quality SAR datasets became available to the public. Noteworthy datasets include OpenSARship [36], a SAR ship detection dataset comprising 11,346 slices, and the HRSID [27] dataset, which consists of 5604 high-resolution ship profile slices. Additionally, datasets with subdivided instance categories have emerged, such as SRSDD [37], which features six categories with 2884 hull instances. The recent MSAR [18] dataset consists of 28,449 detection slices covering four target categories: aircraft, tanks, ships and bridges. This dataset provides a reference for SAR multi-target detection efforts. OEGR-DETR [13] proposed a SAR vehicle detection dataset, which provides researchers with new detection categories. Notably, existing studies on SAR object detection primarily focus on ships, potentially overlooking the significance of multi-category SAR object detection. Unlike previous research proposals for single-category detection on a single dataset, we amalgamated data from various open-source datasets to construct a large SAR object detection dataset for the experiments. The joint dataset encompassed two categories: ships and aircraft. Furthermore, we conduct experiments on a multi-class MSAR dataset to further validate the effectiveness of the proposed method.

## 3. Algorithm Framework

### 3.1. Overall Framework

**Figure 2:**
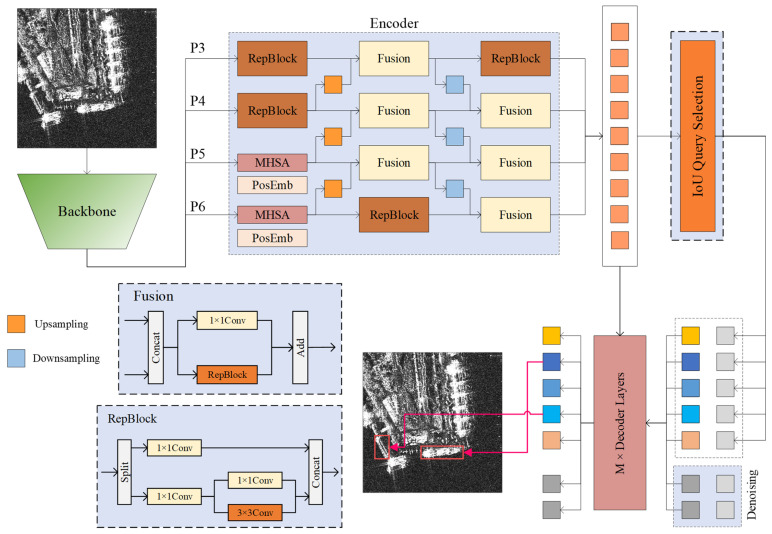
Overall framework of CCDN-DETR. The red box area represents the predicted ship position by the model.

The structure of the CCDN-DETR algorithm is depicted in Figure 2, with the shaded areas representing our proposed improvements. Our method comprises three essential components: the main feature extraction network, the encoder, and the decoder. To maximize the performance of the proposed method, we employed HGNetV2 [38] as the backbone feature extraction network, utilizing the outputs of the last four stages (P3, P4, P5, and P6) as inputs for the cross-scale encoder. Here, P6 represents the downsampled features from the fourth stage of the backbone network. Unlike a traditional DETR encoder, a cross-scale encoder executes a single multi-scale information encoding operation. Subsequently, the denoising decoder selects the top N values with the maximum response from the encoder output vector as the initial input for iterative query optimization. Throughout this process, constrained contrastive denoising was employed for auxiliary training to aid the model in identifying small-scale features. Notably, the output associated with the denoising component in the decoder does not participate in the predictive inference of the model. 

### 3.2. Cross-Scale Encoder Design

The experimental findings from Efficient-DETR [39] reveal that increasing the number of coding layers yields a limited improvement in model accuracy but incurs a substantial computational cost. The feature extraction and information fusion process of the DETR encoder aligns with that of a multiscale pyramid (FPN). Building on this insight, RT-DETR [40] proposed a single-layer hybrid encoder to expedite model inference, providing inspiration for our approach. 

**Figure 3:**
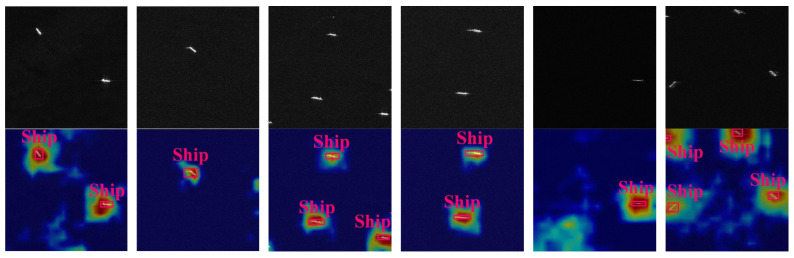
Visualization of the encoder’s area of interest. The upper half of the figure is the original SAR image, and the lower half shows the region of interest of the encoder, the deeper the red region the stronger the model’s attention to the location. Visualization results show that our proposed cross-scale encoder can do a good job of localizing the initial screening of instance targets, helping the decoder to better predict the position of each object.

As illustrated in Figure 2, the proposed cross-scale encoder receives feature information from the four levels of the backbone network. In contrast to the classical FPN [41] structure, the input features of the cross-scale encoder originate from a deeper layer of the backbone network, specifically, the outputs of the second, third, and fourth stages, along with the downsampled information from the fourth stage. This strategic choice contributes to more stable predictions. The cross-scale encoder executes attention and convolution operations separately for deep and shallow semantic features of the input. Utilizing multihead self-attention with location-coded information on deeper high-level features assists the model in capturing long-range information dependencies between instances, enhancing the accuracy of the localization information output. The lower-left portion of Figure 2 shows our Fusion and RepBlock modules designed for efficient feature fusion. For shallow features, additional convolutional blocks continue the feature extraction process. The upsampling layer sequentially fuses the semantic information from the deep layers to the shallow layers, and the layers that have not performed feature fusion are used for the convolution operation using a RepBlock. The fusion of the downsampling layer is similar, and an additional convolution operation is performed at the P3 layer. The cross-scale coder delivers stable and reliable instance features to the decoder layer. Figure 3 shows a heat map of the region of interest of the cross-scale coder, demonstrating its effective focus on objects at different scales.

### 3.3. Query Selection Mechanism Optimization

In the original DETR framework, object queries are initialized as learnable embedding vectors with a value of zero and are iteratively optimized by the decoder. However, because there is no direct correlation between the image features obtained by the encoder and the value of the object query, this setup leads to redundant training time, and the decoder is insufficient to find the optimal solution to the query. In Deformable DETR [42], the features corresponding to the K highest classification scores from the encoder output serve as the initialization reference points for the decoder. Anchor DETR [43] introduced the concept of anchors from CNN detectors, providing explicit physical meaning to instance queries and directing the attention of object queries around the anchor points to enhance performance. DINO DETR [25] proposed a hybrid query approach that initializes the location query using only the top K features and maintains the initialization independence of the content query, thereby filtering out irrelevant information. However, the straightforward strategy of selecting features based on high classification scores for query initialization has limitations. A high classification score for a given object does not guarantee accurate detection, and instances in which high classification scores coexist with low IoU values may exist. To address this concern, we imposed constraints on the queries of the decoder during the model training phase. Specifically, integrating IoU scores into the classification loss optimization process guides the model to prioritize features with both high classification and IoU scores. The classification loss criterion function is defined as in Equation (1):

(1) $$L\mathrm{oss}=-\sum_{i}\left(\alpha \cdot {\left(1-S_{i}\right)}^{\gamma }\cdot \mathrm{IoU}\left(P_{i},G_{i}\right)\cdot \mathrm{CE}\left(S_{i},y_{i}\right)\right)$$

In this formula, $S_{i}$ represents the classification confidence of the predicted bounding box $P_{i}$, $y_{i}$ is the ground truth label, and CE$\left(S_{i},y_{i}\right)$ is the cross-entropy loss function. IoU$\left(P_{i},G_{i}\right)$ is the Intersection over Union (IoU) value between the predicted box $P_{i}$ and the ground truth box $G_{i}$. α and γ are hyperparameters set to 0.25 and 2, respectively, used to adjust the focus of the loss function. Overall, Formula (1) calculates the loss for all predicted bounding boxes in a batch, taking into account both the accuracy of classification and the IoU values between the predicted boxes and the ground truth boxes. The formula reduces the loss contribution of easily classified samples through the focusing factor ${\left(1-S_{i}\right)}^{\gamma }$ and increases the loss weight for accurately localized predicted boxes through the IoU value, thereby optimizing the model’s performance in object detection tasks. 

### 3.4. Denoising Decoder

SAR images encounter challenges such as a high signal-to-noise ratio and blurred object boundary contours, which complicate SAR object detection. To enhance object localization and address these challenges, we introduced a denoising decoder aimed at expediting model training and improving bounding box regression accuracy. The denoising decoder consists of multiple decoding layers and introduces constrained contrast denoising to assist in training.

#### 3.4.1. Constrained Contrastive Denoising Training

DN-DETR considers the randomness of Hungarian matching as an important factor affecting the convergence speed of DETR. The variability in ground-truth assignments by Hungarian matching during different training epochs may introduce information perturbations, hindering the convergence speed. To mitigate this issue, DN-DETR introduces denoising operations during the training process. This involves adding noise to the ground-truth values, with the expectation that the decoder can denoise during the output process and accurately query the true values of the input. It is noteworthy that the denoising training of DN-DETR [35] lacks the ability to discern whether objects exist around anchor points. To address this limitation, DION-DETR [25] introduced contrastive denoising training. This approach employs hyperparameters to control the amount of noise variation, constraining both the true values and noise signals within a defined range. Each set of contrastive denoising includes both positive and negative samples, effectively suppressing redundant queries and facilitating the selection of accurate anchor points for bounding box regression. However, in DINO-DETR, there is a potential confusion between positive and negative samples. This is due to the fact that the range of positive sample bounding boxes is restricted to between 0 and λ. As a result, very small positive samples that do not correspond to the real values at specific anchor points may be activated during training, causing interference. 

Our method involves simultaneously constraining the maximum and minimum variations of both positive and negative samples. The noisy positive and negative samples used for training are derived from variations of the real samples to a certain extent. For the width and height of positive sample bounding boxes, we aim for variations within 50% of the width and height of the real samples. For the width and height of negative sample bounding boxes, we want the variation to be between 50% and 100% of the width and height of the real samples, while ensuring that the smallest bounding box of the negative samples is not less than 10% of the real sample size. We want the noisy positive and negative samples to have no significant variation from the real samples, which will help the network better restore the original coordinates of the instance boxes [15]. For positive samples, the decoder learns the corresponding real sample boxes, while negative samples represent “non-existent objects”.

**Figure 4:**
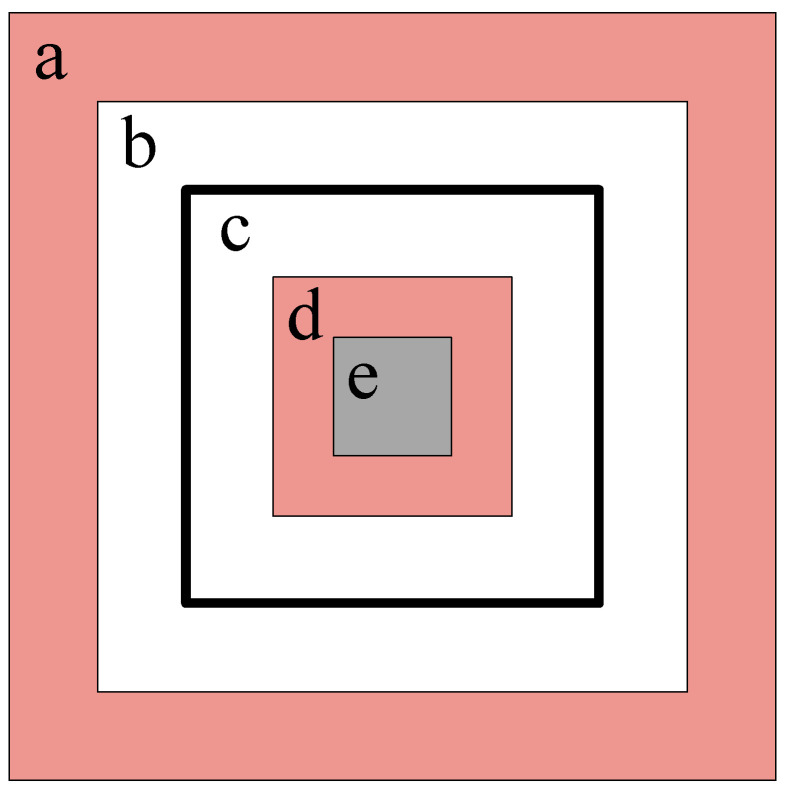
In this figure, the rectangles are defined as a to e based on their size, from largest to smallest. For ease of understanding, let us assume that the true bounding box of an object is a square, represented by rectangle c in this figure. The range of variation for the positive sample bounding boxes is then constrained between rectangles b and d, while the range for negative sample boxes extends from rectangle a to b and from rectangle d to e. That is, the white area represents the range of variation for positive sample boxes, the red area is the range for negative samples, and the gray area is not selected. We further restrict the range of negative samples to encourage the network to learn more accurate positional information.

The training process of contrastive denoising with constraints is divided into several groups: For each group of inputs, n real values are noisified into n positive samples and n negative samples, and then the DINO-DETR training strategy is used. For the positive samples of real values during training, IoU loss and focal loss are used for refinement, while negative samples are categorized as background and optimized using focal loss. The definition of positive and negative samples during the constrained contrast denoising training process is shown in Figure 4.

#### 3.4.2. Decoder Layers

**Figure 5:**
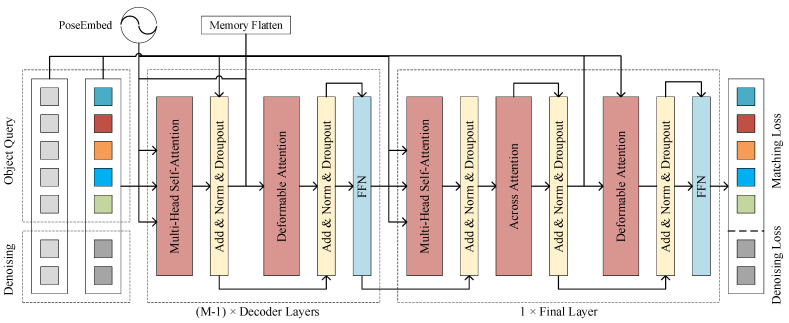
Proposed decoder architecture. The proposed decoder structure, as depicted in Figure 6, comprises attention components, including multihead self-attention and deformable attention. Position encoding is employed to encourage the model to focus on selected spatial positions, and the Memory Flatten operation denotes the flattening of vectors from the encoder output. In CCDN-DETR, a total of M (M = 6) decoding layers have been configured.

As illustrated in Figure 5, the decoder of CCDN-DETR comprised six layers. Each layer consists of multihead self-attention, deformable attention, and regular FeedForward Network (FFN) modules. In the last layer of the decoder, an AcrossAttention [44] module is incorporated to enhance the query selection. The input to the multihead self-attention is derived from the query selected via top-K and localized to the region of interest through position encoding. Deformable attention continues to perform object query operations using key information from the output features of the encoder guiding the query. During the training period, the model underwent constrained contrast denoising operations. Both the positive and negative samples were fed into successive attention modules along with a learnable query. The decoder endeavors to adjust positive samples to their true values while recognizing negative samples as lacking object-related information. Two variants of multihead self-attention were employed in the decoder. Q, K, and V in the AcrossAttention module originate from the query itself, prompting the model to focus on more precise regions. Simultaneously, the deformable attention effectively handles the multi-scale features introduced by the convolutional structure. The attention module concentrates on a subset of sampled points around the reference point and assigns a fixed number of key-assisted queries to each query. This expedites model convergence and mitigates the computational costs associated with a high feature resolution. Deformable attention is expressed as follows:

(2) $$A_{mlqk}\;=\;\mathrm{softmax}\left(\mathrm{AttentionWeights}\left(z_{q}\right)\right)$$

Equation (2) represents the attention weight calculation, where $A_{mlqk}$ represents the attention weight of the mth head, lth level, query position q, and sampling position k, and AttentionWeights represents the application of the attention weight calculation to the query tensor $z_{q}$.

(3) $$\phi_{l}\left(\hat{p_{q}}\right)\;+\;\Delta \;p_{mlqk}\;=\;\mathrm{SamplingLocations}\left(\hat{p_{q}},\;\mathrm{SamplingOffsets}\left(z_{q}\right)\right)$$

Equation (3) represents the sampling location calculation, $\phi_{l}\left(\hat{p_{q}}\right)\;+\;\Delta \;p_{mlqk}$ represents the sampling location at the lth level, normalized reference point p and sampling offset k, while SamplingLocations denotes the calculation of the sampling location based on the normalized reference point and sampling offset.

(4) $$W_{m}^{\prime }=\mathrm{ValueWeights}\left(z_{q}\right)$$

In Formula (4), w represents the weight of the mth head.

(5) $$x_{\mathrm{sampled}}\;=\;\mathrm{GridSampleValues}\left(x^{l},\;\phi_{l}\left(\hat{p_{q}}\right)\;+\;\Delta p_{mlqk}\right)$$

Equation (5) represents grid sampling of the value tensor to obtain sampled values.

(6) $$Deformable\;Attention\;=\;\sum_{m=1}^{M}\;W_{m}\;\left[\;\sum_{l=1}^{L}\;\sum_{k=1}^{K}\;A_{mlqk}\cdot W_{m^{\prime }}\;x_{\mathrm{sampled}}\;\right]$$

Deformable attention produces the final output by linearly combining values from various levels, heads, and sampling positions using attention and value weights within a multihead self-deformable attention mechanism.

## 4. Experiments and Results

### 4.1. Experimental Setup

#### 4.1.1. Dataset

An increasing number of open-source SAR object detection datasets have been proposed, which have significantly contributed to the progress in this field. However, the existing SAR datasets suffer from the problems of single type and small data volume, which are not conducive to the research work. Our scheme unites several available datasets, assembles them into a large SAR object detection benchmark, and conducts experiments in the hope that our approach can provide new ideas for subsequent work. The dataset used as an experiment in this paper consists of SSDD, HRSID, and SAR-AIRcraft, and is called the joint dataset, which consists of two types of objects (boat and aircraft), a total of 11,130 images, and 35,772 instance annotations. We set the ratio of the training, validation, and test sets to 7:1:2. A brief description of the dataset is provided below.

SSDD: SSDD is a SAR ship detection dataset consisting of 1160 annotated images with 2358 ship instances. The images were derived from RadarSat-2, TerraSAR-X, and Sentinel-1 satellites, with each slice image having a size of 500 × 500 pixels.

HRSID: HRSID was derived from Sentinel-1B, TerraSAR-X, and TanDEM-X satellite imagery. Originally consisting of 136 panoramic SAR images, they were divided into 5604 image slices of 800 × 800 pixels each. This dataset contained 16951 MS COCO-type ship annotations.

SAR-AIRcraft: SAR-AIRcraft is a SAR aircraft object detection dataset with a single polarized cluster beam imaging mode. There were 4368 images and 16,463 aircraft annotations, including common carriers such as the A320 and Boeing737, with an image resolution of up to 1500 × 1500.

In addition, we report the performance of CCDN-DETR on the MSAR dataset, which is a challenging large-scale benchmark. The MSAR dataset uses data from both the Haisi-1 and Gaofen-3 satellites, covering a variety of complex scenarios, such as airports, harbors, coasts, islands, and urban areas, and includes four categories, namely aircraft, tanks, bridges, and ships, with 28,449 data slices and 60,396 labelled instances. We also set the ratio of training, validation, and test data to 7:1:2.

#### 4.1.2. Training Settings

The CCDN-DETR was trained on a single RTX3090 using CUDA 11.6, CUDNN 8.5, and PyTorch 1.13.1. The training batch size was set to 8, with a total of 72 training epochs. Optimization was performed using the Adam optimizer with an initial learning rate of 1 × 10^−4^. The backbone network had an initial learning rate of 1 × 10^−5^, with the learning rate diminishing to 1/10 of the original rate at the 36th epoch. The input image size was uniformly set to 640 × 640, and standard data augmentations were applied, including random rotation (−45° to 45°), random scale scaling (0.75 to 1.25), random cropping, and color distortion, following the practices outlined in PP-YOLOE [45]. The initialization involved 100 vector features from the encoder outputs, and the decoder was configured with six layers. The experimental setup described above also applies to ablation experiments unless otherwise stated.

### 4.2. Evaluation Metrics

To fully demonstrate the performance of the proposed CCDN-DETR, we used precision, recall, mAP50, and mAP50-95 as evaluation metrics. Precision is the ratio of the number of samples correctly identified as positive to the total number of samples identified as positive by the model, and recall is the ratio of the number of samples correctly identified as positive to the total number of samples in the positive class. The formulas for precision and recall are as follows:

(7) $$Precision=\frac{True\;Positives}{True\;Positives+False\;Positives}$$

(8) $$Recall=\frac{True\;Positives}{True\;Positives+False\;Negatives}$$

where True Positives represent the number of samples correctly identified as positive classes, False Positives represent the number of samples incorrectly identified as positive classes (misclassifying the negative class samples as positive), and False Negatives represent the number of samples incorrectly identified as positive classes.

In object detection, the mean Average Precision (mAP) was used to evaluate the performance of the detection model. The calculation of mAP involves precision–recall curves (PR curves) and the computation of the area under these curves (AUC). mAP50 specifically refers to the calculation of the mAP using an IoU threshold of 0.5. The steps for computing mAP50 are as follows:

(9) $$\mathrm{mAP}50=\frac{1}{N}\sum_{i=1}^{N}\left(\sum_{n}\left(R_{n}-R_{n-1}\right)\times P_{n}\right)$$

where N is the total number of target categories, and $R_{n}$ and $P_{n}$ denote the points on the interpolated precision–recall curve for recall and precision, respectively.

### 4.3. Experiment Results

To demonstrate the effectiveness of our methodology, we provide a performance comparison between our approach and benchmark methods for both the test and validation sets. The benchmark models include YOLO V5 [21], YOLO V8, RetinaNet [6], RTMDet [46], and Faster R-CNN [2] and transformer-based models such as DINO-DETR [25], Deformable DETR [30], RT-DETR [36], and CO-DETR [47].

#### 4.3.1. Comparison on the Joint Dataset

Table 1 and Table 2 present the comparative experimental results for the joint dataset’s validation and test sets, respectively. For the validation set, CCDN-DETR exhibits superior performance when compared to existing DETR models. Our method achieves an 11.4-point increase in the mAP50-95 metric compared to Deformable DETR, with only a 0.1 M increase in the number of parameters. Versus DINO-DETR, it gains a higher recall rate (an increase of 4.8) and a 5.3-point improvement in the mAP-95 metric, further demonstrating the effectiveness of our proposed constrained contrastive denoising. Additionally, CCDN-DETR achieves performance that is on par with advanced CNN models. With 2.2 M fewer parameters than YOLO V8, it manages to improve mAP50-95 by 1.2 points, further illustrating the advantages of self-attention in long-distance modeling. 

**Figure 6:**
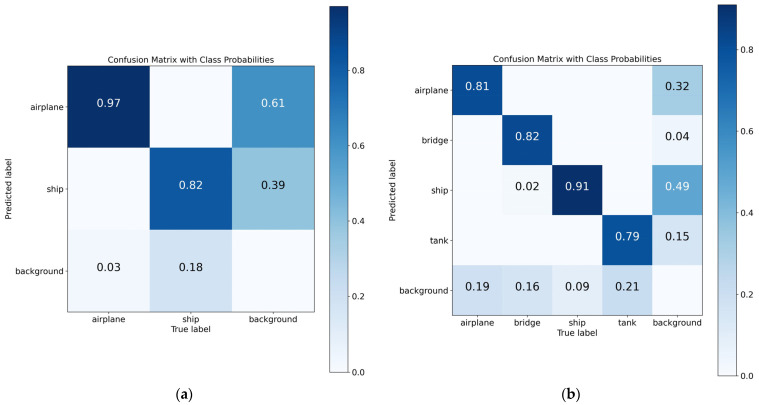
Performance of CCDN-DETR in multi-category SAR object detection. Our method demonstrates outstanding performance in multi-category object detection, achieving high detection accuracy in the joint dataset. Moreover, in the MSAR dataset, the accuracy of detecting ships, tanks, and bridges is notably high. (**a**) Joint dataset. (**b**) MSAR.

In the comparative experimental results on the validation set with more data, CCDN-DETR still maintains excellent performance. With 8.3 M fewer parameters than RT-DETR, it improves mAP50-95 by 2.0 points; compared to CO-DETR, it increases the recall rate by 4.6 and mAP by 2.2, demonstrating the stability of the framework we proposed. Compared to the CNN-based model RTMDet, mAP50-95 improves by 2.6, with only a 0.3-point decrease in mAP50, indicating that CCDN-DETR performs better in scenarios with higher precision (IoU threshold) requirements. Figure 6a shows the confusion matrix of CCDN-DETR on the joint dataset validation set, from which it can be seen that our method has a good effect on recognizing ships, especially airplanes.

#### 4.3.2. Comparison on the MSAR Dataset

The MSAR dataset consists of four categories and has a larger amount of data with denser samples, which presents a significant challenge for object detection tasks. Table 3 and Table 4, respectively, present the comparative experimental results on the MSAR dataset’s validation and test sets. On the validation set, CCDN-DETR still maintains a high level of detection performance. With 6.7 M fewer parameters than CO-DETR, it increases mAP50 by 2.6, indicating that our method performs better in dense scenes; similarly, compared to RT-DETR, CCDN-DETR has 8.5 M fewer parameters and increases mAP50-95 by 2.2, demonstrating that our proposed constrained contrastive denoising module performs well in detecting small SAR objects. Compared to the state-of-the-art CNN model YOLO v8, CCDN-DETR has 2.2 M fewer parameters and increases mAP50-95 by 0.3, with only a 0.6-point decrease in mAP50, suggesting that our method can more accurately localize object coordinates. Figure 6b shows the confusion matrix of our method on the validation set, indicating that CCDN-DETR has good classification performance in aircraft and bridges, especially in the ship category, and only slightly worse in dense tank scenes.

In the test set with more data and more instances, CCDN-DETR still maintains good performance. Its parameter count is 1 M less than DINO-DETR, and the mAP50-95 metric increases by 7.3, indicating that our method has higher accuracy under stringent testing conditions and demonstrating the significant gains of the cross-scale encoder and constrained contrastive denoising in complex scenarios. Compared to the latest CNN model RTMDet, although our method lags behind by 1.4 in recall rate, it improves by 1.6 in the mAP50-95 metric, which requires higher precision, showcasing the advantage of the transformer in long-distance modeling. Additionally, some methods like Deformable DETR, RetinaNet, and Faster R-CNN perform slightly worse, and even Deformable DETR lags behind Faster R-CNN by 2.3 in mAP50. This may be due to the lack of relevant denoising training in Deformable DETR, leading to poorer performance in the more challenging dataset.

### 4.4. Ablation Experiments

In this section, we conducted a detailed analysis of the modules and configurations influencing the performance of CCDN-DETR. Specifically, we explored the impact of the denoising training module, query selection method in the input decoder, and the number of layers in the decoder. Additionally, we report a comparison between the performance gains of cross-scale encoders and multi-layer encoders, as well as a comparison between CCDN-DETR and classical object detection algorithms in terms of inference speed. Comparative experiments were conducted using the validation set of the joint dataset, maintaining consistency with the experimental settings outlined in Section 4.1.2.

#### 4.4.1. Denoising Training Module Ablation Experiments

This section discusses the impact of denoising training modules. Initially, we established a baseline by removing the denoising module from CCDN-DETR. Subsequently, we introduced and evaluated three denoising modules: the DN denoising module, DINO denoising module (CDN), and our proposed constrained contrast denoising module. Table 5 illustrates the effect of the denoising modules on the model performance. The accuracy of the baseline without denoising training was reduced compared to CCDN-DETR, with a 4.8-point reduction in the mAP50 metric and a 5.3-point reduction in the more stringent mAP50-95 metric. This shows that denoising training can significantly improve model performance. Additionally, CCDN-DETR exhibits a slight performance advantage over DINO, which is attributed to the more precise restrictions on noise addition in our approach, facilitating a more efficient localization of complex targets. By incorporating contrastive denoising during training, CCDN-DETR constrains noise to an appropriate range, allowing the decoder to intuitively capture potential true samples and enhance the focus of the model on small objects.

#### 4.4.2. Impact of Query Initialization and Quantity

**Figure 7:**
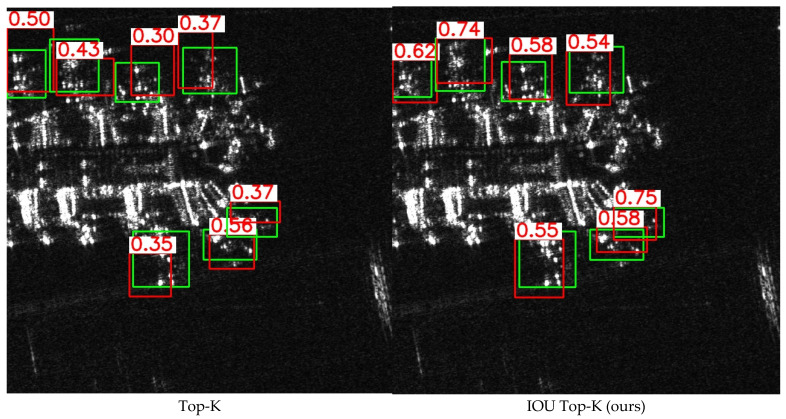
Differences in the initial position features selected by different query initialization approaches. The basic query initialization approach focuses only on features with high classification scores and does not necessarily select features with high IOU scores well, which may cause some interference to the subsequent decoder in finding the optimal solution to the query. Our approach guides the model to select both coder features with high classification scores and high IOU scores, which helps the model to further localize the object.

The choice of initialization method and quantity of queries significantly influence DETR’s performance of DETR. We compared the fundamental approach of selecting the top K-class maximum values for initialization with the CCDN-DETR method, which incorporates an IOU score constraint during initialization. Additionally, we varied the value of K, setting it to 100 or 300, to examine the effect of the number of queries on model accuracy. The results, as presented in Table 6, demonstrate that the proposed query initialization scheme achieves a greater improvement in accuracy. However, the use of more queries did not significantly enhance the CCDN-DETR. This was attributed to the limited occurrence of multiple objects in the same image within the training data, rendering 100 queries sufficient to cover most of the SAR test scenarios. Figure 7 illustrates the disparities between the initial position queries selected using the proposed method and the basic query initialization approach. The proposal to use IoU thresholds as weights can effectively guide the model to select features with both high classification scores and high IoU scores, thereby enhancing the model’s localization capability.

#### 4.4.3. Decoder Layer Quantity Selection

In most DETR studies, the decoder is usually set to six layers, which is considered to satisfy the requirement of model accuracy and ensure that the scale of the model is not too large. In this section, we set CCDN-DETR to different numbers of decoder layers to observe its effect on the results; the results are shown in Table 7. We find that for decoder layers up to six, the model performance gradually increases with the number of decoders, and the inference time consumption increases in parallel. A higher number of decoder layers has extremely limited gain for the model, but instead brings huge inference time consumption as well as larger memory overhead, which is inappropriate for the application level. Therefore, we chose six layers of decoders as the solution for the CCDN-DETR scheme.

#### 4.4.4. Encoder Selection

In this section, we delve into the performance gains achieved by varying encoder configurations while keeping the rest of the model’s architecture constant. We focus on reporting the impact of changes solely in the encoder configuration on the model’s performance. We used traditional multi-layer encoders and our proposed cross-scale encoder as comparison benchmarks. For the traditional encoder, we selected 3-layer and 6-layer encoding setups for comparative analysis. Table 8 shows the influence of different encoder configurations on model performance. It can be seen that our proposed cross-scale encoder has a significant performance improvement over the classic multi-layer encoder, achieving a 2.4-point increase in the mAP50-95 metric compared to the 6-layer encoder and a 3.4-point increase compared to the 3-layer encoder. Additionally, even though the multi-layer encoder significantly reduces the number of parameters (by 5.6 M), it results in an additional 5.8 ms of inference time. We attribute this efficiency improvement to the cross-scale encoder’s adoption of fewer self-attention modules, while the cross-scale feature fusion promotes information interaction and enhances model performance. The cross-scale fusion coding strategy employed in CCDN-DETR facilitates feature fusion across different scales, striking a balance between the inductive bias of convolution and the long-range modeling capability of self-attention.

#### 4.4.5. Comparison of Inference Speed

In this section, we will conduct a comparative analysis of the inference speeds among different models, including CCDN-DETR, Deformable DETR, DION-DETR, as well as YOLO V8 and Faster-RCNN based on CNN models. We randomly selected 500 images from the validation set of the joint dataset for speed testing and calculated the average. The batch size of the inference phase is set to 1, and the measured time includes data preprocessing and model inference time.

Table 9 illustrates the time consumed by different models for inference on a single image. It can be observed that CCDN-DETR exhibits significant advantages in terms of inference speed and accuracy compared to the DETR family of models. Compared to Deformable DETR, CCDN-DETR achieves an 11.4-point improvement in mAP50-95 with only a 2 ms increase in inference latency. Versus DION-DETR, it improves by 5.3 points in mAP50-95 and reduces latency by 5.6 ms, thanks to the efficient structural design of the proposed cross-scale encoder. Additionally, compared to CNN-based models, CCDN-DETR does not have an advantage in terms of inference speed due to its complex structure and the frequent memory access required by multihead self-attention operations. Although it achieves a 20-point improvement in mAP50-95 compared to Faster R-CNN, the latency nearly doubles. Compared to the state-of-the-art YOLO V8, CCDN-DETR’s inference latency increases by 19.4 ms. For the entire DETR series of models, how to accelerate inference is a question worth researching.

### 4.5. Visualization

In this section, we present the visual results of CCDN-DETR for the detection of four classes: ships, aircraft, bridges, and tanks. To demonstrate the effectiveness of our method, we provide visual comparisons with ground truth (GT) annotations, as well as detection results from Faster R-CNN for reference.

**Figure 8:**
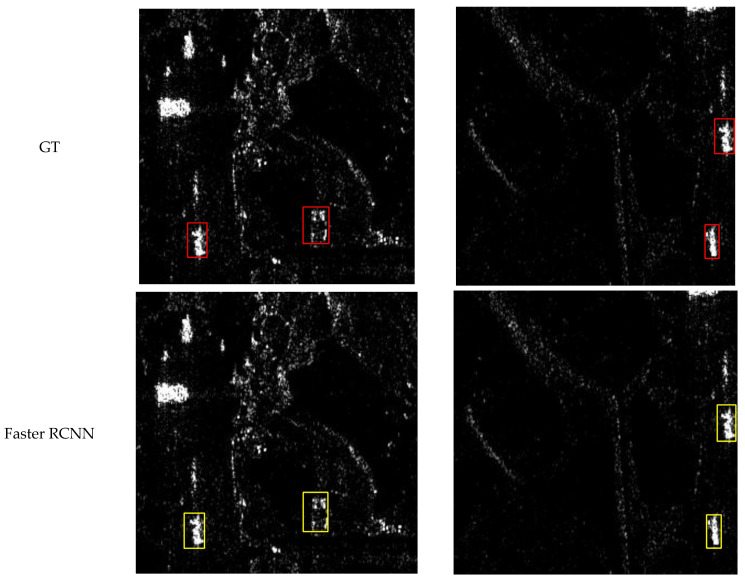

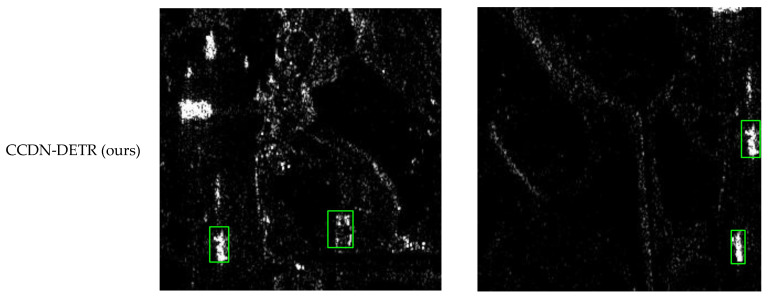
Detection comparison in SAR ship recognition scene.

**Figure 9:**
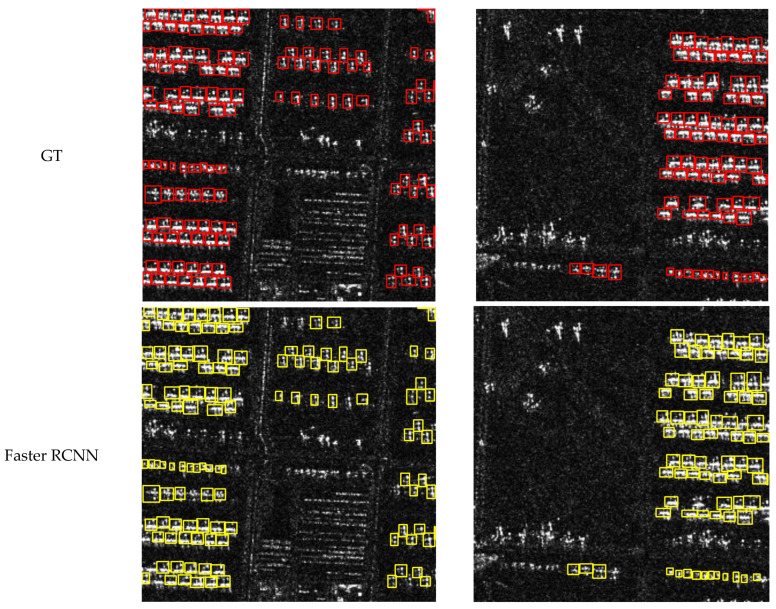

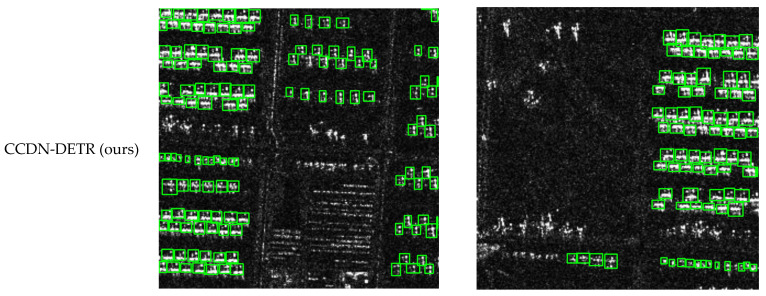
Detection comparison in SAR aircraft recognition scene.

**Figure 10:**
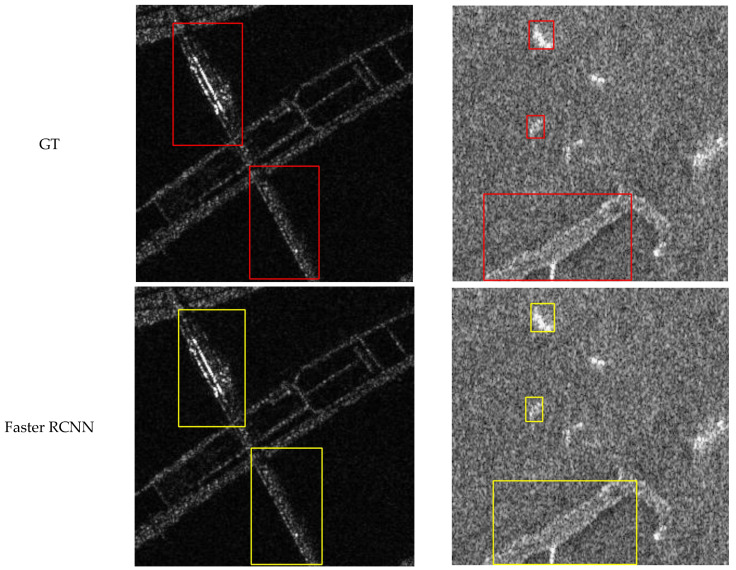

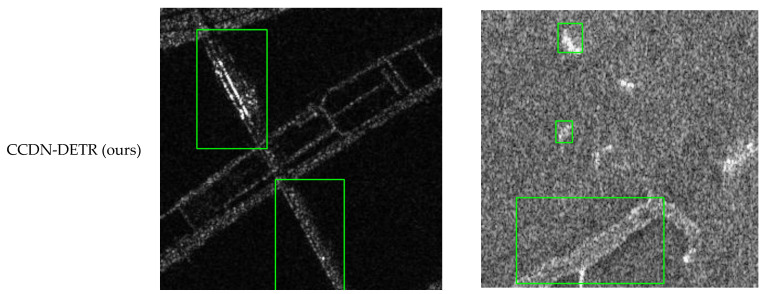
Detection comparison in SAR bridge recognition scene.

**Figure 11:**
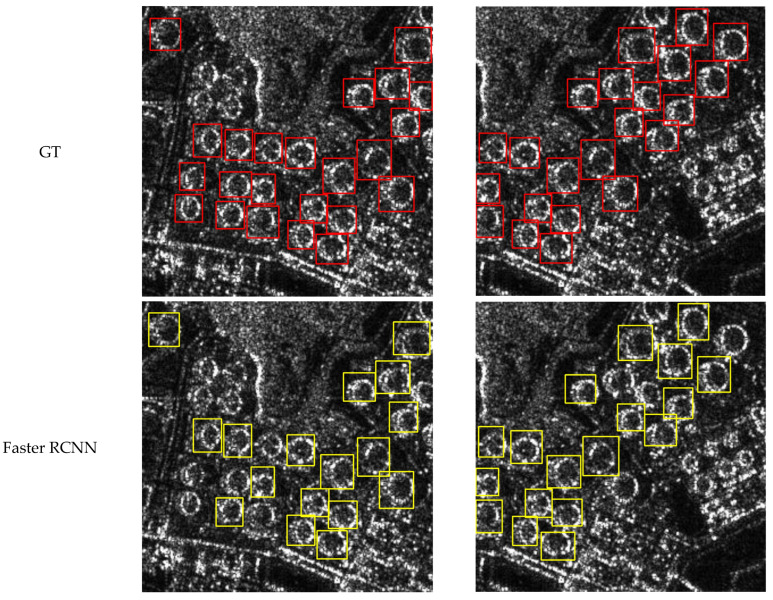

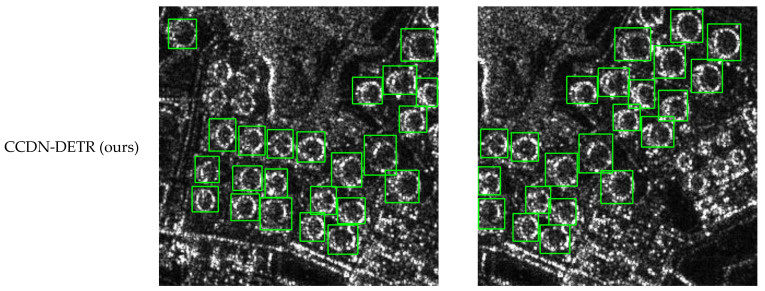
Detection comparison in SAR tank recognition scene.

Figure 8, Figure 9, Figure 10 and Figure 11 shows the visualization results for a scenario involving ships, aircraft, bridges, and tanks. The red box represents the real annotation box, the yellow box represents the prediction result of Faster R-CNN, and the green box represents the prediction result of CCDN-DETR. It is evident that CCDN-DETR performs well in most scenarios. In the ship detection scenario, CCDN-DETR exhibits higher precision in the predicted bounding boxes, whereas the Faster R-CNN results show greater perturbations. The airplane detection scenario is particularly complex, with smaller target areas and multiple instances within a single image. The compared methods all exhibit some degree of incomplete recognition. However, CCDN-DETR ensures that the detected boxes have higher accuracy while identifying more objects. The bridge recognition scenario is relatively simple, with all selected methods guaranteeing efficient identification. In the tank detection scenario, which is slightly different due to a larger number of instances and individual targets, CCDN-DETR demonstrates outstanding detection performance, while Faster R-CNN occasionally misses targets.

Overall, CCDN-DETR achieved a detection accuracy comparable to that of advanced CNN detectors, owing to the effectiveness of our proposed approach.

## 5. Discussion

**Figure 12:**
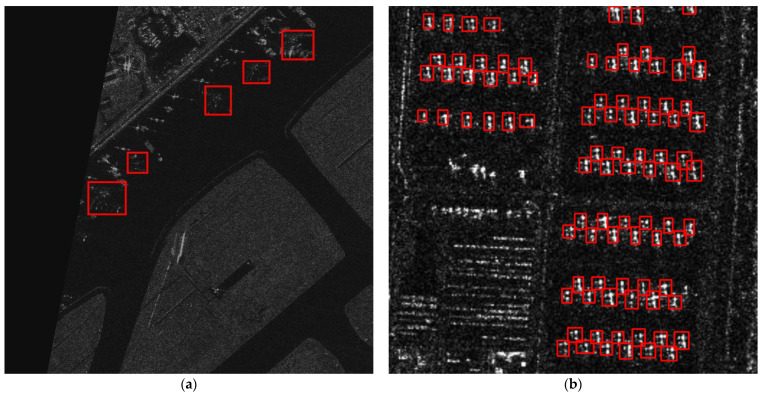
Different imaging of the airplane between the different datasets. The red areas represent examples of airplanes. (**a**) Airplane in the SAR-AIRcraft dataset. (**b**) Airplane in the MSAR dataset.

In fact, the idea of merging datasets adopted in this paper is not the first time it has been implemented. There have been some works [49,50,51] that proposed merging multiple SAR datasets for experiments and achieved good results. It is well known that deep learning training requires a substantial amount of data. Using only a small amount of data for experiments may lead to overfitting, where the model performs well only on the small dataset but poorly in a new real-world scenario. Additionally, due to the lack of convolutional inductive bias in transformers, their training requires more high-quality data compared to CNN models, which is also one of the reasons for merging datasets. Using a large amount of training data not only effectively addresses overfitting but also enhances the model’s generalization ability, allowing it to perform better in completely new SAR detection scenarios. However, it is important to note that not all SAR data are suitable for merging in experiments. We need to consider the imaging conditions between different datasets and the degree of resolution difference between images, as shown in Figure 12, where (a) shows the aircraft model in the SAR-AIRcraft dataset, while (b) shows the aircraft model in the MSAR dataset. There is a significant difference in imaging conditions and a large disparity in image resolution (the resolution of SAR-AIRcraft images is 1500 × 1500, while the MSAR images are only 256 × 256), making them unsuitable for merging datasets for experimentation. It is hoped that some of our discussions can provide insights for future work.

## 6. Conclusions

This paper introduces CCDN-DETR, an end-to-end framework designed for synthetic aperture radar (SAR) multi-class object detection. The method replaces the original multi-layer transformer encoder with a cross-scale encoder and proposes an IoU loss for query selection optimization. A more powerful decoder layer is designed, and the noise box generation scheme for constrained contrastive denoising is optimized, significantly improving the performance of the DETR model in the field of SAR target detection. Experimental results on two different datasets show that the performance of CCDN-DETR can rival popular CNN frameworks. This highlights the tremendous potential of the DETR framework in the challenging field of SAR object detection.

Our experimental results indicate that the encoder structure of the DETR framework can perform feature extraction using a hybrid of CNNs and transformers, and the introduction of CNN’s inductive bias ability helps the model accurately locate SAR targets. For the inherent crowded scene characteristics of SAR images, selecting queries entering the decoder reasonably can further enhance model performance. The proposed scheme incorporating IoU loss helps the decoder select queries with more precise localization and performs optimization. Additionally, due to the small size and concentration of SAR targets, introducing denoising training is crucial for improving DETR’s detection of SAR targets. It is necessary to adopt constrained contrastive denoising to strengthen the model’s ability to handle small objects.

## Figures and Tables

**Table 1:** Comparison on the joint dataset val set.

Method	Inference	Backbone	Parameters (M)	Precision	Recall	mAP50	mAP50-95
YOLO V5 [21]	ultralytics	CspDarkNet	25.1	0.849	0.827	0.893	0.606
YOLO V8	ultralytics	CspDarkNet	25.8	0.873	0.849	0.918	0.623
RetinaNet [6]	ICCV2017	ResNet-FPN	19.8	0.786	0.757	0.879	0.418
RTMDet [46]	MMDetection	CspNext	24.7	0.871	0.850	0.926	0.606
Faster R-CNN [2]	NeurIPS2015	ResNet-FPN	28.3	0.744	0.739	0.879	0.435
Deformable DETR [42]	ICLR2021	ResNet [48]	23.5	0.835	0.809	0.867	0.521
DINO-DETR [25]	ICLR2023	ResNet [48]	24.6	0.837	0.808	0.913	0.582
RT-DETR [40]	PaddlePaddle	HGNetV2	31.9	0.864	0.848	0.911	0.619
CO-DETR [47]	ICCV2023	ResNet	30.1	0.870	0.837	0.905	0.622
CCDN-DETR (ours)	/	HGNetV2	23.6	0.891	0.856	0.919	0.635

**Table 2:** Comparison on the joint dataset test set.

Method	Inference	Backbone	Parameters (M)	Precision	Recall	mAP50	mAP50-95
YOLO V5 [21]	ultralytics	CspDarkNet	25.1	0.834	0.812	0.881	0.612
YOLO V8	ultralytics	CspDarkNet	25.8	0.870	0.852	0.925	0.629
RetinaNet [6]	ICCV2017	ResNet-FPN	19.8	0.776	0.752	0.878	0.414
RTMDet [46]	MMDetection	CspNext	24.7	0.871	0.849	0.925	0.605
Faster R-CNN [2]	NeurIPS2015	ResNet-FPN	28.3	0.743	0.738	0.878	0.431
Deformable DETR [42]	ICLR2021	ResNet [48]	23.5	0.830	0.808	0.865	0.517
DINO-DETR [25]	ICLR2023	ResNet [48]	24.6	0.836	0.806	0.912	0.580
RT-DETR [40]	PaddlePaddle	HGNetV2	31.9	0.856	0.825	0.904	0.611
CO-DETR [47]	ICCV2023	ResNet	30.1	0.846	0.828	0.900	0.608
CCDN-DETR (ours)	/	HGNetV2	23.6	0.887	0.874	0.922	0.631

**Table 3:** Comparison on MSAR dataset val set.

Method	Inference	Backbone	Parameters (M)	Precision	Recall	mAP50	mAP50-95
YOLO V5 [21]	ultralytics	CspDarkNet	25.1	0.835	0.783	0.839	0.571
YOLO V8	ultralytics	CspDarkNet	25.8	0.837	0.788	0.843	0.579
RetinaNet [6]	ICCV2017	ResNet-FPN	19.8	0.665	0.592	0.570	0.345
RTMDet [46]	MMDetection	CspNext	24.7	0.835	0.780	0.835	0.564
Faster R-CNN [2]	NeurIPS2015	ResNet-FPN	28.3	0.741	0.656	0.708	0.435
Deformable DETR [42]	ICLR2021	ResNet [48]	23.5	0.756	0.643	0.682	0.437
DINO-DETR [25]	ICLR2023	ResNet [48]	24.6	0.835	0.712	0.808	0.493
RT-DETR [40]	PaddlePaddle	HGNetV2	31.9	0.838	0.772	0.832	0.559
CO-DETR [47]	ICCV2023	ResNet	30.1	0.831	0.745	0.811	0.527
CCDN-DETR (ours)	/	HGNetV2	23.6	0.834	0.788	0.837	0.581

**Table 4:** Comparison on MSAR dataset test set.

Method	Inference	Backbone	Parameters (M)	Precision	Recall	mAP50	mAP50-95
YOLO V5 [21]	ultralytics	CspDarkNet	25.1	0.819	0.772	0.829	0.573
YOLO V8	ultralytics	CspDarkNet	25.8	0.825	0.784	0.841	0.577
RetinaNet [6]	ICCV2017	ResNet-FPN	19.8	0.648	0.602	0.563	0.342
RTMDet [46]	MMDetection	CspNext	24.7	0.825	0.789	0.832	0.568
Faster R-CNN [2]	NeurIPS2015	ResNet-FPN	28.3	0.742	0.665	0.702	0.427
Deformable DETR [42]	ICLR2021	ResNet [48]	23.5	0.760	0.655	0.679	0.441
DINO-DETR [25]	ICLR2023	ResNet [48]	24.6	0.830	0.718	0.805	0.511
RT-DETR [40]	PaddlePaddle	HGNetV2	31.9	0.837	0.769	0.832	0.559
CO-DETR [47]	ICCV2023	ResNet	30.1	0.820	0.757	0.804	0.529
CCDN-DETR (ours)	/	HGNetV2	23.6	0.845	0.775	0.829	0.584

**Table 5:** Effect of denoising training module on model performance.

Method	Precision	Recall	mAP50	mAP50-95
BaseLine	0.839	0.818	0.871	0.582
BaseLine (+DN)	0.865	0.819	0.894	0.594
BaseLine (+CDN)	0.884	0.832	0.912	0.622
CCDN-DETR (ours)	0.891	0.856	0.919	0.635

**Table 6:** Impact of query’s selection scheme on model performance.

Query Selection	Precision	Recall	mAP50	mAP50-95
Top-K (K = 100)	0.877	0.843	0.905	0.625
Top-K (K = 300)	0.874	0.845	0.903	0.626
IOU Top-K (K = 100)	0.891	0.856	0.919	0.635
IOU Top-K (K = 300)	0.894	0.851	0.922	0.638

**Table 7:** Impact of decoder layer quantity on model performance and inference speed.

Number of Decoder Layers	Precision	Recall	mAP50	mAP50-95	Parameters (M)	Latency (ms)
1	0.833	0.818	0.794	0.600	17.8	27.4
3	0.878	0.846	0.901	0.621	20.1	29.9
6	0.891	0.856	0.919	0.635	23.6	34.3

**Table 8:** Impact of encoder on model performance.

Encoder Selection	Precision	Recall	mAP50	mAP50-95	Parameters (M)	Latency (ms)
Multi-layer encoder (3 layers)	0.849	0.816	0.899	0.601	15.5	32.2
Multi-layer encoder (6 layers)	0.875	0.840	0.907	0.611	17.9	40.1
Cross-scale encoder	0.891	0.856	0.919	0.635	23.6	34.3

**Table 9:** Inference time test.

Method	Precision	Recall	mAP50	mAP50-95	Parameters (M)	Latency (ms)
Faster-RCNN	0.744	0.739	0.879	0.435	28.3	17.5
YOLO V8	0.873	0.849	0.918	0.623	25.8	14.9
Deformable DETR	0.835	0.809	0.867	0.521	23.5	32.4
DION-DETR	0.837	0.808	0.913	0.582	24.6	39.9
CCDN-DETR	0.891	0.856	0.919	0.635	23.6	34.3

## Data Availability

The data used in this study are open datasets. Data can be obtained at https://github.com/da13132/joint_dadatset_of_SAR (Accessed on 28 February 2024).
